# Supplementary material for: Towards women-inclusive ecology: Representation, behavior, and perception of women at an international conference
Source: PLoS One. 2021 Dec 10;16(12):e0260163. doi: 10.1371/journal.pone.0260163 (PMC8664204; doi:10.1371/journal.pone.0260163)
Supplement: S1 Appendix — (PDF) [file pone.0260163.s001.pdf]

TEMPLATE FOR OBSERVERS

Session:

Date:

Time:

Speaker's Name:

BLOCK 1. GENERAL INFORMATION

Question	Answer	Description
**Observer**		Your name. This info will not be shared, it is only for queries.
**Speaker_Sex**	**MALE / FEMALE**	Gender of the speaker.
**Chair_Sex**	**MALE / FEMALE**	Gender of the chair that introduced the speaker.
**Talk_Topic**	**F / T / M / TH / E**<br>**Bg / Pop / Bio / Others**	Topic of the talk. **Options:** Freshwater (F) / Marine (M) / Terrestrial (T) / Theoretical (TH) / Evolution & Ethology (E) / Biogeochemistry (Bg) / Populations (Pop) / Biodiversity (Bio) / Others
**Talk_Start_Ok**	**YES / NO**	The talk started on time.
**Talk_Duration**		Duration of the talk in minutes
**Chair_Intervention**	**YES / NO**	The chair intervened because the speaker exceed his/her time.

BLOCK 2. ATTENDANCE INFORMATION

Includes the attendances, the chairs, and the observer (i.e. you), but not the speaker. Please, be accurate. Count at least twice for each case (and more times if you have disagreement!). Avoid counting during the first or last minutes as people is usually coming in and out of the room (e.g. count the attendees between slide 5 and 6).

Question	Answer	Description
**Numer_Male**		Nmber of male attendees
**Number_Female**		Number of female attendees
**Number_Attendees**		Number of total attendees

BLOCK 3. QUESTION TIME

Question	Answer	Description
**Silence_Period**	**YES / NO**	There was a silence period after the talk.
**Silence_Duration**		Duration of the silence period in seconds
**Chair_Question**	**YES / NO**<br>**FIRST / LAST / UNIQUE**	The chair asked a question. If yes, specify if it was the first, last or unique question.
**No_Question**	**YES / NO**	No questions were asked in this talk.
**1_Question_Sex**	**MALE / FEMALE**	Gender of the attendee that asked the question
**1_Question_Duration**		Duration of the question in seconds
**1_Answer_Duration**		Duration of the answer in seconds
**2_Question_Sex**	**MALE / FEMALE**	Gender of the attendee that asked the question
**2_Question_Duration**		Duration of the question in seconds
**2_Answer_Duration**		Duration of the answer in seconds
**3_Question_Sex**	**MALE / FEMALE**	Gender of the attendee that asked the question
**3_Question_Duration**		Duration of the question in seconds
**3_Answer_Duration**		Duration of the answer in seconds
**4_Question_Sex**	**MALE / FEMALE**	Gender of the attendee that asked the question
**4_Question_Duration**		Duration of the question in seconds
**4_Answer_Duration**		Duration of the answer in seconds
**5_Question_Sex**	**MALE / FEMALE**	Gender of the attendee that asked the question
**5_Question_Duration**		Duration of the question in seconds
**5_Answer_Duration**		Duration of the answer in seconds
**6_Question_Sex**	**MALE / FEMALE**	Gender of the attendee that asked the question
**6_Question_Duration**		Duration of the question in seconds
**6_Answer_Duration**		Duration of the answer in seconds
