# Supplementary material for: Towards women-inclusive ecology: Representation, behavior, and perception of women at an international conference
Source: PLoS One. 2021 Dec 10;16(12):e0260163. doi: 10.1371/journal.pone.0260163 (PMC8664204; doi:10.1371/journal.pone.0260163)
Supplement: S2 Appendix — Note that we only present selected results here. (PDF) [file pone.0260163.s002.pdf]

Informed Consent Statement

You are invited to participate in a research study examining the role of men and women and their visibility during the SIBECOL 2019 meeting. In addition, we will ask you to answer some basic demographic questions. This survey will take 10-15 minutes to complete.

This research study is organized by the Gender group of the Iberian Association of Limnology (AIL) and has the support of the Iberian Ecological Society (SIBECOL) board. If you have any questions about the study, you may contact genderscienceail@gmail.com.

This survey is anonymous, and your responses will be kept completely confidential. No personally identifiable data will be collected and no one will be able to link your answers back to you.

Participation in this research is completely voluntary. If you decide to participate, you may withdraw at any point during the study, for any reason, and without any prejudice. By clicking the "I consent" button below, you acknowledge that your participation in the study is voluntary, you are 18 years of age or older, and that you are aware that you may choose to terminate your participation in the study at any time and for any reason.

- ○ I consent
- ○ I do not consent, I do not wish to participate

Q1. Did you present any scientific contribution as first author at the conference?

- ○ Yes, poster presentation
- ○ Yes, 15 minutes oral presentation
- ○ Yes, 30 minutes keynote presentation
- ○ Yes, plenary talk
- ○ No

Q2. During oral communications, did you always asked a question when you wanted to?

- Yes
- No, sometimes I had questions that I did not ask
- I did not ask questions because I did not want to

Q3. At the conference, who asked more questions during oral communications, women or men?

- Women asked more questions
- Men asked more questions
- Women and men asked about the same amount of questions
- I do not know

Please, indicate how much do you agree with the following statements.

Q4. During the conference, others came to me to discuss intellectual ideas

- Strongly disagree
- Disagree
- Neither agree nor disagree
- Agree
- Strongly agree

Q5. During the conference, I suffered the impostor syndrome, i.e. felt like a fraud or like you are not as competent as others perceive you to be

- ◯ Strongly disagree
- ◯ Disagree
- ◯ Neither agree nor disagree
- ◯ Agree
- ◯ Strongly agree

Q6. I think the conference organization was supportive of colleagues who want to balance their work and personal life

- ◯ Strongly disagree
- ◯ Disagree
- ◯ Neither agree nor disagree
- ◯ Agree
- ◯ Strongly agree

Q7. I am satisfied with the amount of social interactions with others at the conference

- ◯ Strongly disagree
- ◯ Disagree
- ◯ Neither agree nor disagree
- ◯ Agree
- ◯ Strongly agree

Q8. I am satisfied with the level of intellectual stimulation at the conference

- ◯ Strongly disagree
- ◯ Disagree
- ◯ Neither agree nor disagree
- ◯ Agree
- ◯ Strongly agree

Q9. In general, I liked attending the conference

- ◯ Strongly disagree
- ◯ Disagree
- ◯ Neither agree nor disagree
- ◯ Agree
- ◯ Strongly agree

Q10. During the conference, to what extent did you feel excluded from social activities?

- ◯ Never
- ◯ Rarely
- ◯ Occasionally
- ◯ Frequently
- ◯ Very Frequently

Q11. During the conference, to what extent did someone pay little attention to your statement or show little interest in your opinion?

- ◯ Never
- ◯ Rarely
- ◯ Occasionally
- ◯ Frequently
- ◯ Very Frequently

Q12. During the conference, to what extent did someone put you down or was mean to you?

- ◯ Never
- ◯ Rarely
- ◯ Occasionally
- ◯ Frequently
- ◯ Very Frequently

Q13. During the conference, to what extent did you hear any gender stereotypical remark?

- ◯ Never
- ◯ Rarely
- ◯ Occasionally
- ◯ Frequently
- ◯ Very Frequently

Q14. During the conference, to what extent did you confront someone who made a sexist comment or behaved in a sexist way?

- ◯ Never
- ◯ Rarely
- ◯ Occasionally
- ◯ Frequently
- ◯ Very Frequently

Q15. During the conference, to what extent did you remain silent in response to sexist comments or behaviors?

- ◯ Never
- ◯ Rarely
- ◯ Occasionally
- ◯ Frequently
- ◯ Very Frequently

You indicated that you contributed as a speaker at the SIBECOL conference. Please, indicate how much do you agree with the following statements.

Q16. When choosing the members of the audience, I think the moderator of my session was gender biased

- ○ Strongly disagree
- ○ Disagree
- ○ Neither agree nor disagree
- ○ Agree
- ○ Strongly agree

Q17. The questions were constructive

- ○ Strongly disagree
- ○ Disagree
- ○ Neither agree nor disagree
- ○ Agree
- ○ Strongly agree

Q18. The questions were formulated politely

- ○ Strongly disagree
- ○ Disagree
- ○ Neither agree nor disagree
- ○ Agree
- ○ Strongly agree

Q19. I felt satisfied with my answers to questions

- ○ Strongly disagree
- ○ Disagree
- ○ Neither agree nor disagree
- ○ Agree
- ○ Strongly agree

Please, answer the following information about yourself (mark the appropriate answers)

Q20. What is your age?

▼ 18 ... +70

Q21. What is your gender?

- ○ Female
- ○ Male
- ○ Different gender identity (please specify)

____

Q22. Do you belong to any underrepresented minority group? Select all that qualify.

- ☐ Race / ethnic minority
- ☐ LGBT (lesbian, gay, bisexual, transgender)
- ☐ People with disabilities
- ☐ Other (please specify): ____
- ☐ None

Q23. Which is the country of your nationality?

▼ Afghanistan ... Zimbabwe

Q24. In which country are you currently living?

▼ Afghanistan ... Zimbabwe

Q25. In which career stage are you?

- ○ Undergraduate or Master Student
- ○ PhD Candidate / Pre-doctoral Researcher
- ○ Early Career Researcher / Post-doctoral Researcher
- ○ Senior Professor/Researcher - Non-permanent position
- ○ Senior Professor/Researcher - Permanent position
- ○ Other (please specify): ____

Q26. In which type of institution do you develop your research?

- ◯ University
- ◯ Research Institution
- ◯ Public Agency / Public Administration
- ◯ Private Company
- ◯ Non-Governmental Organization (NGO)
- ◯ Unemployed

Q27. Please, write here any other comment regarding this survey that you would like us to know

____
