# Supplementary material for: Towards women-inclusive ecology: Representation, behavior, and perception of women at an international conference
Source: PLoS One. 2021 Dec 10;16(12):e0260163. doi: 10.1371/journal.pone.0260163 (PMC8664204; doi:10.1371/journal.pone.0260163)
Supplement: S3 Appendix — It includes S1 and S2 Tables. (PDF) [file pone.0260163.s003.pdf]

**S3 Appendix.** Summary of the collected data of "Towards women-inclusive ecology: Representation, behavior, and perception of women at an international conference".

**Table S1.** Number of presenters at the 1st SIBECOL Meeting, classified by gender and contribution type.

| | Gender | Pre-doctoral | Post-doctoral | Senior non-permanent | Senior permanent | Other |
|---|---|---|---|---|---|---|
| ***Presenters*** | Female | 107 | 82 | 33 | 56 | 8 |
| | Male | 88 | 76 | 39 | 83 | 1 |
| ***Posters*** | Female | 58 | 8 | 5 | 16 | 7 |
| | Male | 29 | 19 | 9 | 19 | 0 |
| ***Oral*** | Female | 49 | 72 | 26 | 33 | 1 |
| | Male | 59 | 55 | 27 | 50 | 1 |
| ***Invited*** | Female | 0 | 1 | 1 | 5 | 0 |
| | Male | 0 | 2 | 1 | 11 | 0 |
| ***Plenary*** | Female | 0 | 1 | 1 | 2 | 0 |
| | Male | 0 | 0 | 2 | 3 | 0 |

**Table S2.** Comparison of audience behavior during talks given by women and men. Variables are: number of attendees (average ± standard error) for each contribution type; questions received (total number and standardized by number of talks per gender), total number of questioners (classified by gender) and total number of first questioners (classified by gender).

| | **Female Speaker** | **Male Speaker** |
|---|---|---|
| **Attendees** | | |
| Oral | 39 ± 5 | 43 ± 4 |
| Invited | 46 ± 8 | 50 ± 7 |
| Plenary | 239 ± 76 | 282 ± 29 |
| **Questions received** | | |
| Total number of questions | 162 | 178 |
| Standardized by the no. of talks per gender | 1.37 | 1.51 |
| **Questioners** | | |
| Female | 71 | 66 |
| Male | 91 | 112 |
| **First questioner** | | |
| Female | 31 | 32 |
| Male | 48 | 60 |
