# Supplementary material for: Towards women-inclusive ecology: Representation, behavior, and perception of women at an international conference
Source: PLoS One. 2021 Dec 10;16(12):e0260163. doi: 10.1371/journal.pone.0260163 (PMC8664204; doi:10.1371/journal.pone.0260163)
Supplement: Attachment — Submitted filename: Message to the Authors.docx [file pone.0260163.s004.docx]

*Message to the Authors:*

This review is for the manuscript, “Towards women-inclusive ecology: Representation, behavior, and perception of women at an international conference” (PONE-D-21-16326). The authors investigated the attendance and participation (as both speakers and audience members) of women at the 1^st^ Meeting of the Iberian Society of Ecology, as well as potential differences in perception of inclusion and community between female and male participants. Through documentation of the invited speaker panels, the authorship of research presentations, and the gender of those asking questions following research presentations, as well as a post-meeting survey, the authors documented a relative lack of female last authors (signifying a lack of female-led research groups presenting) as well as a lack of female invited keynote speakers. Women also appeared to ask fewer questions and, from the post-meeting survey, feel less included and free to ask questions than their male peers. Results varied based on professional demographics (e.g., early career vs. permanently positioned later career participants), with those women who had “stuck it out” responding in surveys that they felt free to ask questions and felt professionally satisfied and included at the meeting. These results suggest that professional meetings should evaluate methods for inviting speakers and including the entire community in question and answer/discussion times to better accommodate all attendees. Although PLoS ONE does not evaluate manuscripts based on their novelty or marketability, this manuscript contributes toward a better understanding of how women interact within the academic environment and suggests several paths toward better inclusion, which, as the authors note, is critical to preventing professional stagnation and loss of women from the academic ecological workforce. Analyses were appropriately performed, and the manuscript is technically sound. I have a few concerns regarding the conclusions drawn from the data, but with revisions to better clarify what conclusions can be drawn from the self-selected survey as well as how global the application of these results may be, given the focus of the conference being the Iberian Peninsula, I believe this research is scientifically sound and makes a meaningful contribution to our knowledge.

Attached are the comments on major and minor issues I would recommend be addressed. I want to be very clear that none of the below issues reflect my personal opinion on or experience with gender discrimination, but are questions and concerns that occurred to me regarding the data and conclusions presented.

*Major Issues*

- 32% of attendees completed the survey, and 60% of those were women, which is significantly different from the composition of attendees. Further discussion of the potential influence of self-selection in responding to the survey is warranted. The authors question the self-reporting of women in the discussion, further calling into question the survey results. This needs to be addressed.
- I am concerned about the temporal aspect of the conclusions regarding last authorship. If it takes a significant amount of time to rise to the level of PI that would produce a large quantity of research, how can we conclude that the lack of female last authorship at this conference isn’t a product of conditions that discouraged female participation at upper-level research institutions a decade ago, rather than some factor of invitation or current environment? The same concerns would arise for conference organizers and committee leaders. These positions require time to achieve, so I do wonder whether this is a bias or a lack of women at high-level (i.e., R1) institutions that have the funding and time to guide, support and participate in these roles, and whether the early-career female attendees signal any movement away from this situation. I would like to see some discussion of this possibility.
- 83% of respondents were of Spanish nationality, and most were living in Spain during the time of the conference. While the conference was international, I do wonder how cultural differences may play a role in the global applicability of the results. While I do not suspect that the overall conclusions and recommendations should be altered, I think this is a point worth discussing, particularly regarding the cultural perceptions of women in the workforce, domestic obligations, parenthood, familial caregiving, and other factors that vary heavily across cultures.
- The results in Table 2 show statistically significant differences in feeling imposter syndrome and hearing gender stereotypical remarks. While these differences are statistically significant, are the differences significant in the real world, or perhaps an artifact of how survey respondents perceive frequency, potentially based on sensitivity to an event? The numbers for imposter syndrome (Q5) for both women and men would fall in “fairly frequent” or “middle of the road”, while gender stereotypical remarks (Q11) would fall on the “next-to-never” end of the spectrum for both men and women. Are either of these factors, then, truly underlying differences in conference participation?
- Please clarify what specific aspects of Q&A sessions favor men. This topic is mentioned several times in the discussion but is never stated outright in terms of what the authors mean.

*Minor Issues*

- Lines 89-91: should be “This theory states…”; this description of the theory is also unclear and should be clarified.
- Lines 173-174: this is vague.
- Line 184: should be “latter”
- Lines 340-341: how many more comments, on average?
- Lines 348-349: “bias” can imply intentional unfairness. Unless there is evidence of this, I would consider rewording.
- Line 388: remove “to”
- Lines 391-392: is there some evidence that we can educate our way out of this kind of subtle inequitable situation? Recommendations are given for how to alter conference format, but this suggests that there may be a place for formal education. It seems a bit open here for additional comment/discussion.
- Lines 398-399: the results do not suggest that any action be taken. Is there any evidence that we shouldn’t teach women scientists to assert themselves more aggressively during Q&A? What specific structures and dynamics currently favor men? If the end goal is to increase women’s participation, what has been shown to work? This sentence either needs to be elaborated on with citations or re-worded.
- Line 403: remove “to”
- Line 403-405: Line 406 says that this sentence is speculative. Once you question this result, many other possibilities regarding self-selection/self-reporting within your survey crop up. Perhaps remove speculation entirely or devote a paragraph to exploring the possibilities of this self-selection/self-reporting. It’s likely that men self-report differently, too.
- Lines 424-425: Among those who responded to the survey.
- Line 427-430: should be “self-report”; also, there is the possibility that women who encountered sexist attitudes were more likely to respond to the survey.
- Line 435: Please define earlier what specific dynamics of a Q&A are favorable to men. Also, is the exclusion of women from social events direct or indirect?
- Line 462: what kind of clear guidelines and criteria? Are there any that have shown promise, such as a quota system, or are there other frameworks?
- Line 480: should this be “to ask” rather than “to think”? Also, while I think turn taking has promise, forcing attendees to participate seems aggressive to me, in that some attendees may prefer to just listen and learn at their first conference or at a conference on a subject they are just beginning to explore.
- Lines 489-491: citations needed
