# Supplementary material for: Towards women-inclusive ecology: Representation, behavior, and perception of women at an international conference
Source: PLoS One. 2021 Dec 10;16(12):e0260163. doi: 10.1371/journal.pone.0260163 (PMC8664204; doi:10.1371/journal.pone.0260163)
Supplement: Attachment — Submitted filename: Response to Reviewers.docx [file pone.0260163.s005.docx]

**Reviewer #1**

*This review is for the manuscript, “Towards women-inclusive ecology: Representation, behavior, and perception of women at an international conference” (PONE-D-21-16326). The authors investigated the attendance and participation (as both speakers and audience members) of women at the 1st Meeting of the Iberian Society of Ecology, as well as potential differences in perception of inclusion and community between female and male participants. Through documentation of the invited speaker panels, the authorship of research presentations, and the gender of those asking questions following research presentations, as well as a post-meeting survey, the authors documented a relative lack of female last authors (signifying a lack of female-led research groups presenting) as well as a lack of female invited keynote speakers. Women also appeared to ask fewer questions and, from the post-meeting survey, feel less included and free to ask questions than their male peers. Results varied based on professional demographics (e.g., early career vs. permanently positioned later career participants), with those women who had “stuck it out” responding in surveys that they felt free to ask questions and felt professionally satisfied and included at the meeting. These results suggest that professional meetings should evaluate methods for inviting speakers and including the entire community in question and answer/discussion times to better accommodate all attendees.*

*Although PLoS ONE does not evaluate manuscripts based on their novelty or marketability, this manuscript contributes toward a better understanding of how women interact within the academic environment and suggests several paths toward better inclusion, which, as the authors note, is critical to preventing professional stagnation and loss of women from the academic ecological workforce. Analyses were appropriately performed, and the manuscript is technically sound. I have a few concerns regarding the conclusions drawn from the data, but with revisions to better clarify what conclusions can be drawn from the self-selected survey as well as how global the application of these results may be, given the focus of the conference being the Iberian Peninsula, I believe this research is scientifically sound and makes a meaningful contribution to our knowledge.*

*Attached are the comments on major and minor issues I would recommend be addressed. I want to be very clear that none of the below issues reflect my personal opinion on or experience with gender discrimination, but are questions and concerns that occurred to me regarding the data and conclusions presented.*

**Answer:** Thank you for your positive feedback and constructive criticism. We agree with the reviewer that some of our conclusions were rather speculative and we rewrote the discussion and conclusions accordingly. Please, see our responses to your comments for detailed information about those changes.

**Major Issues**

*32% of attendees completed the survey, and 60% of those were women, which is significantly different from the composition of attendees. Further discussion of the potential influence of self-selection in responding to the survey is warranted. The authors question the self-reporting of women in the discussion, further calling into question the survey results. This needs to be addressed.*

**Answer:** The reviewer is right; there is a mismatch in the proportion of women that responded the survey vs those that attended the conference. This pattern in the response rates is common in gender studies, as women tend to participate more because they are more aware of and sensitive to gender issues compared to men [Carter et al. 2018; Biggs et al. 2017; Slauson-Blevins and Johnson, 2016]. Further, as the reviewer noticed, there is no way to know whether our results represent the perception of all attendees or are skewed towards those who responded the survey (i.e. people who are more aware of gender issues). However, it is impossible to test for the occurrence of this phenomenon and, if it occurred, it is then applicable for both women and men. In any case, we want to notice that the response rate for both genders (40% of female attendees, 24% of male attendees) was large enough for statistical analyses, and higher than those observed in other conferences (typically from 10 to 20%) [Cook et al. 2000; Biggs et al. 2017]. Hence, we feel confident of our results. Following the reviewer suggestion, we added this rationale in the methods (lines 218-219, 231-234).

On the other side, we agree with the reviewer that we should not question the responses to our survey because it was speculative and inappropriate. Following their suggestion, we removed those sentences (line 406 of the former manuscript). Now, we do not question the responses. Thanks for noticing.

*I am concerned about the temporal aspect of the conclusions regarding last authorship. If it takes a significant amount of time to rise to the level of PI that would produce a large quantity of research, how can we conclude that the lack of female last authorship at this conference isn’t a product of conditions that discouraged female participation at upper-level research institutions a decade ago, rather than some factor of invitation or current environment? The same concerns would arise for conference organizers and committee leaders. These positions require time to achieve, so I do wonder whether this is a bias or a lack of women at high-level (i.e., R1) institutions that have the funding and time to guide, support and participate in these roles, and whether the early-career female attendees signal any movement away from this situation. I would like to see some discussion of this possibility.*

**Answer:** We agree that the reduced number of women as keynote speakers, conveners and last authors might be a product of conditions that discouraged female participation at upper-level research institutions in the past. In our manuscript, this phenomenon is called “demographic inertia”. However, we do not believe that the demographic inertia alone can explain the observed results because we actually found a great proportion of females (>40%) within senior researchers registered in the conference, which indicates that high-level institutions in the Iberian Peninsula had both women and men within their academic staff. Therefore, we argue that other factors, including the implicit bias, a lower visibility of women in science, or the tendency of women to decline invitations because of higher share of domestic tasks, might also influence differences in gender representation. We added this rationale in the discussion (lines 377-383). Further, we clarified the definition of “demographic inertia” in the introduction (lines 89-94).

*83% of respondents were of Spanish nationality, and most were living in Spain during the time of the conference. While the conference was international, I do wonder how cultural differences may play a role in the global applicability of the results. While I do not suspect that the overall conclusions and recommendations should be altered, I think this is a point worth discussing, particularly regarding the cultural perceptions of women in the workforce, domestic obligations, parenthood, familial caregiving, and other factors that vary heavily across cultures.*

**Answer:** It is right; the cultural perceptions of women might vary among cultures. The culture of the Iberian Peninsula is European and it represents the situation of women in most First World countries [Poushter and Fetterolf 2019]. For instance, in Spain, there is a good preconception of women as scientists that is like those reported in France, Germany and UK [L’Oréal survey, 2015]. Also, the proportion of women working in science in Spain and Portugal (>40% of the scientists) is higher than the world’s average (33%) and similar to other European countries. Therefore, we argue that our research stresses the importance of advancing towards more women-inclusive conferences even in First World countries. This rationale was included in the last section of the discussion (lines 462-462).

*The results in Table 2 show statistically significant differences in feeling imposter syndrome and hearing gender stereotypical remarks. While these differences are statistically significant, are the differences significant in the real world, or perhaps an artifact of how survey respondents perceive frequency, potentially based on sensitivity to an event? The numbers for imposter syndrome (Q5) for both women and men would fall in “fairly frequent” or “middle of the road”, while gender stereotypical remarks (Q11) would fall on the “next-to-never” end of the spectrum for both men and women. Are either of these factors, then, truly underlying differences in conference participation?*

**Answer:** We agree that most respondents (60%) reported to have never heard stereotypical remarks during the conference, and that none of them reported to have heard stereotypical remarks very frequently. However, we found that 43% of women reported to have heard some stereotypical remarks, while only 34% of the men did. Moreover, more female than male respondents reported to have heard stereotypical remarks occasionally or frequently (17% vs. 6%). These results indicate that either women are the target of most of these stereotypical remarks or that they are more aware of them. In either case, we believe that these differences are important because they underlie a distinct perception of conferences. Also, we believe that the accumulation of “rarely” and “occasionally” heard stereotypical remarks makes women feeling less accepted within the scientific community. We have added new results (lines 348-355) and discussion (lines 434-439) on this regard.

Similarly, it is right that most respondents did not agree nor disagree with the question “I suffered the impostor syndrome”. Indeed, only 28% of the respondents agreed or strongly agreed to have suffered the impostor syndrome during the conference. However, this percentage was almost twice higher for women than for men (34% vs 19%), suggesting once again that the impostor syndrome affects more women than men. We have added this rationale in the results (lines 355-357) and discussion (lines 450-460).

*Please clarify what specific aspects of Q&A sessions favor men. This topic is mentioned several times in the discussion but is never stated outright in terms of what the authors mean.*

**Answer:** Current Q&A dynamics favour competition and are usually dominated by those attendees that have more authority or have a close relationship with the convener. These people are generally (but not always) senior male researchers [Carter 2018; Karpowitz 2021; King 2018]. We have modified the text so as to express that is not that Q&A sessions favour men *per se*, but that their format is not very welcoming to women (lines 407-416).

**Minor Issues**

*Lines 89-91: should be “This theory states…”; this description of the theory is also unclear and should be clarified.*

**Answer:** Following the reviewer suggestion, we clarified that the demographic inertia theory states that gender biases in conferences mostly mirror imbalances in gender ratios among senior members, who are the ones that participate the most in these type of event (lines 89-94).

*Lines 173-174: this is vague.*

**Answer:** We clarified that volunteers did not participate in the session they were analyzing nor explain the study to third parts. This procedure allowed us to observe the behavior of the attendees without conditioning it (lines 175-177).

*Line 184: should be “latter”.*

**Answer:** Ok (line 198).

*Lines 340-341: how many more comments, on average?*

**Answer:** Unfortunately, we did not ask for the number of stereotypical remarks they heard, but about their frequency (i.e. never, rarely, occasionally, frequently, or very frequently; see Q13 of the S2 Appendix). Therefore, we compared the amount of female vs male respondents who self-reported to have listened any stereotypical remark. We found that 43% of women self-reported to have heard some stereotypical remarks, while only 34% of the men did. Moreover, more female than male respondents reported to have heard stereotypical remarks occasionally or frequently (17% vs. 6%). We added these results in lines 352-355.

*Lines 348-349: “bias” can imply intentional unfairness. Unless there is evidence of this, I would consider rewording.*

**Answer:** The reviewer is right. We changed it to “imbalances” (lines 361).

*Line 388: remove “to”.*

**Answer:** Ok (line 402).

*Lines 391-392: is there some evidence that we can educate our way out of this kind of subtle inequitable situation? Recommendations are given for how to alter conference format, but this suggests that there may be a place for formal education. It seems a bit open here for additional comment/discussion.*

**Answer:** We believe that formal education on diversity and inclusion is essential to build a new equitable system in science. The implementation of mandatory courses within research centers might help to change the attitudes and behaviors of scientists [Chang et al. 2019]. However, the same study suggests that short courses might not be as effective as we believed, and that more effortful interventions, including recruitment of women or the change of processes and institutional structures, may be needed to robustly change people behavior. In any case, we acknowledge that formal education techniques on diversity and inclusion go beyond the scope of our study, which focuses on conferences behaviors. Therefore, we deleted these sentences from the discussion.

*Lines 398-399: the results do not suggest that any action be taken. Is there any evidence that we shouldn’t teach women scientists to assert themselves more aggressively during Q&A? What specific structures and dynamics currently favor men? If the end goal is to increase women’s participation, what has been shown to work? This sentence either needs to be elaborated on with citations or re-worded.*

**Answer:** Currently, “stage-time” dynamics favor competition, and are usually dominated by those attendees that have more authority or have a close relationship with the convener [Carter 2018, Karpowitz 2021, King 2018]. However, previous studies have shown that women are more likely to ask questions if they do not have to compete to be called on, or when the environment is respectful, mindful, and familiar [Telis et al. 2019; Konrad et al. 2000; Bailey et al. 2020]. Accordingly, we found that women self-reported asking questions whenever they wanted less frequently than men at most career stages. The only exception was for senior permanent researchers, where more women than men self-reported to have asked questions whenever they wanted, which might suggests an increased feeling of community belonging of those women that “survived” the leaky pipeline [Ceci et al. 2014]. This rationale is now included in the discussion (lines 407-420).

Further, we now argue that the solution to this imbalance may not be bursting women to behave more aggressively during Q&A times, but rather to rethink the structure and dynamics of Q&A sessions for fostering the participation of all participants. Following the reviewer suggestion, we included specific examples of structures and dynamics that can favor the participation of women during Q&A sessions in the last section of the discussion (lines 493-500). These dynamics include small group discussions, use of technology/social media as support for Q&A session, or speed-dating activities to promote discussion among participants within a more relaxed environment. Also, some recommendations to the conveners on how to deal with timekeeping issues and reformulate misplaced questions might help to establish a more respectful and mindful environment during the sessions.

*Line 403:* *remove “to”.*

**Answer:** Ok (line 420).

*Line 403-405: Line 406 says that this sentence is speculative. Once you question this result, many other possibilities regarding self-selection/self-reporting within your survey crop up. Perhaps remove speculation entirely or devote a paragraph to exploring the possibilities of this self-selection/self-reporting. It’s likely that men self-report differently, too.*

**Answer:** Following the reviewer suggestion, we removed these speculative sentences.

*Lines 424-425: Among those who responded to the survey.*

**Answer:** Right; we now state that “subtle distinct responses between genders also indicate that women tended to have a worse experience than their male counterparts, at least among the survey respondents” (lines 435-436).

*Line 427-430: should be “self-report”; also, there is the possibility that women who encountered sexist attitudes were more likely to respond to the survey.*

**Answer:** Right; we changed it to “self-report”. Further, we added the explanation suggested by the reviewer in the methods (lines 231-234).

*Line 435: Please define earlier what specific dynamics of a Q&A are favorable to men. Also, is the exclusion of women from social events direct or indirect?*

**Answer:** Following the reviewer´s suggestion, we now specify that “stage-time” dynamics favor competition and are usually dominated by those attendees that have more authority or a close relationship with the convener, which are generally men [Carter 2018, Karpowitz 2021, King 2018] (lines 411-413). Further, we now clarify that the exclusion of women from social events is often indirect (lines 446-448). For instance, many after-conference activities compromise the participation of women with family responsibilities.

*Line 462: what kind of clear guidelines and criteria? Are there any that have shown promise, such as a quota system, or are there other frameworks?*

**Answer:** Following the reviewer suggestion, we clarified which guidelines we were referring to. In particular, we suggest that the inclusion of gender quotas, the promotion of female scientist’ directories and the facilitation of formal letters explaining the reasons of choosing a particular keynote speaker should be designed and provided to conference organizers (lines 474-477).

*Line 480: should this be “to ask” rather than “to think”? Also, while I think turn taking has promise, forcing attendees to participate seems aggressive to me, in that some attendees may prefer to just listen and learn at their first conference or at a conference on a subject they are just beginning to explore.*

**Answer:** We agree that forcing attendees to participate can be aggressive and hence, we do not recommend this practice. Instead, we recommend other initiatives, including small group discussions, the use of technology/social media as support for Q&A session, or speed-dating activities to promote discussion among participants within a more relaxed environment. We clarified this issue (lines 493-500).

*Lines 489-491: citations needed.*

**Answer:** Following the reviewer suggestion, we have included examples of codes of conduct (i.e. EGU, SFS) and also citations that support the benefits of supporting child care or promoting safe spaces for women at conferences [Calisi et al. 2018] (lines 505-507).

**Reviewer #2:**

*The paper analyses the representation, behavior and perception of woman at the international conference of the 1st Meeting of the Iberian Society of Ecology. The authors used a novel, multidimensional approach to analyze women’s representation, behavior and perception at a medium sized conference as their study object with a good participation (766 participants at the conference), 56% of the 375 talks analyzed for women’s behavior and 32% of all attendees attending the post-conference survey on their perception.*

*My impression of the manuscript is that the available data is analyzed thoroughly and that the conclusions from the data are applicable. The title reflects the contents of the paper well, without overselling the results. The abstract shows the results and their interpretation in a too simplified way and in my opinion overstates some of the facts (please see my comments under detailed examination) which could lead to a misconception for the reader and should be, in parts, rewritten more carefully. The introduction is concise and gives a good overview over the topic while leading up to the aims of the study. The results are concise and well put together to address the raised questions and the discussion is to the point while giving good explanations of the results. However, in the introduction it was mentioned that the multidimensional approach should be used to detect mechanisms behind the gender bias. I do not think that with the approach used here mechanisms can be detected, thus the explanations given in the discussion are hypothetical (which is correctly stated in the discussion). I think the authors should be clearer here, their analysis shows nicely women’s representation, the audience’s behavior and its perception of this behavior, however, there was no question ask during the study as to the ‘why’ (mechanisms) of certain behavior. I do think however, that the study has merit even, when hypothesizing on these mechanisms, but this should be made clear.*

**Answer:** Thank you for your positive comments. Following the reviewer suggestion, we rewrote the abstract and introduction to avoid misconceptions, especially regarding the objectives of the study. Please, see our responses to your specific comments for more information.

**Detailed examination**

*Line 51: It is true that women might experience sexism at conferences, however, in your case, you stated there was none overtly sexism reported. I find that statement in the first sentence of the abstract misleading for the expectations on the whole paper. I would rather argue that conferences are showing a cross section of the scientific community in a given field and are thus ideal study objects for the analysis of gender imbalances, as you have conducted.*

**Answer:** Following the reviewer suggestion, we rewrote the first sentence of the abstract. It now reads as: “Conferences are ideal platforms for studying gender gaps in science because they are important cultural events that reflect barriers to women in academia” (lines 50-51).

*Line 59: The statements on the possibility of women to ask questions and their intellectual satisfaction are overselling the results in my opinion; i.e. the statement ‘most women were unable to ask the questions they wanted’ implies someone or something prevented them actively from asking a question. While as reported in line 400 (self-report) it says ‘women self-reported to have asked questions whenever they wanted less frequently’ does not imply any active bias against women. Also, although the difference between men and women in their frequency to answer questions whenever they wanted in Fig 5. is significant, it also shows that most men did not always asked questions whenever they wanted either, with only a difference in career stage. This should not be oversimplified and formulated more carefully. The authors stated quite correctly in the discussion, that there are various reasons, e.g. a short time frame for the Q&A sessions, which let to women not asking questions and they also stated that their interpretation of the behavior is speculative. Thus, I think the formulation in the abstract should be more oriented on the actual question which was answered by the participants in the post-conference survey. The same goes for: ‘most women […] showed limited intellectual satisfaction’: in table 1 Q7 of the post-conference survey, woman had a satisfaction value of 4.06 ± 0.80 on a scale of 1-5 while men had a value of 4.16 ± 0.78 and the difference between men and women was not significant. Thus, I think the statement, that ‘the post-conference survey revealed that most women were unable to ask the questions they wanted and showed limited intellectual satisfaction during the conference’ is overselling the results and misleading for the reader and should thus be carefully rewritten.*

**Answer:** The reviewer is right. We rewrote the highlighted sentences to match with the results. We now state that: “The post-conference survey revealed that attendees had a good experience and did not perceive the event as a threatening context for women. Yet, differences in the responses between genders suggest that women tended to have a worse experience than their male counterparts” (lines 58-61).

*Line 113: I do not think that with the studied angles -representation, behavior and perception - can detect mechanisms behind gender bias. I rather think that using a multidimensional approach gives a more comprehensive picture, but to detect mechanisms, questions about the ‘how’ of a certain behavior would need to be addressed.*

**Answer:** The reviewer is completely right. Accordingly, we now state that “our multidimensional approach is critical to provide a comprehensive assessment of the situation of women in sciences, and to develop evidence-based policymaking promoting inclusive scientific conferences for female researchers” (lines 115-117).

*Line 126: (max. one per session).*

**Answer:** Ok, changed (line 128).

*Line 157: true for 97% of cases – how was this tested? Was this part of the survey? Or based on who was first author, versus who presented the study? Did the presenter not need to be first author?*

**Answer:** This percentage was based on who was first author vs who presented the study. Generally, the presenter is the first author, but not always. This last case occurs when, for personal reasons, the first author cannot attend the conference. We clarified this rationale in the methods (lines 160-161).

*Line 178: mismatch observed with [noted?] gender – Could this create a problem in the analysis?*

**Answer:** We agree that not knowing the gender of the questioners and audience might create problems in the analysis. Ideally, all attendees should have self-reported their gender identity and/or pronouns during the registration and identified themselves in each session. However, this was logistically not possible. Therefore, we decided that the less invasive way to report the gender of attendees was to rely on the criteria of the observers. In any case, we do not believe that this methodology influenced our study, as none of the person who answered the post-conference survey self-reported to belong to other genders than male or female.

*183/184: ‘… while the most frequent answer was computed for gender-related questions.’ I do not understand this, please rephrase.*

**Answer:** We meant that, for gender-related questions (i.e., those whose answer was “man” or “woman”), we chose the answer that most observers marked. Note that, for most talks, all observers agreed with the gender of questioners. Mismatches in the gender of questioners only occurred on five occasions (2.3% of cases). We clarified this issue in the methods (lines 186-189).

*Also on line 183: A praise for the study design: if the inter-observer variability was <5%, the protocol was very well thought out.*

**Answer:** Many thanks!

*Line 192: Why were the sessions not independent? Because the audience can be the same between sessions?*

**Answer:** Yes, the audience was free to change from one room to another whenever they wanted. Therefore, the same audience could attend different sessions. We clarified this issue in the methods (lines 196-198).

*Line 360: general sessions were mostly convened by men: Could this be a historical artifact? General sessions are mostly the same each year, thus it could be likely that the conveners are the same each time?*

**Answer:** We agree that, for some conferences, conveners for general sessions are the same persons every year. However, note that this was the first SIBECOL conference ever held and therefore, it was the conference organization who decided all the conveners for general sessions. We clarified this issue in the discussion (lines 369-370).

*Line 377: In the sessions where there were mostly women as conveners there were also more female keynotes: this could also show networks. Conveners are likely to ask people from their own networks to give a keynote speech (know the persons talking style). Thus, if men have a network dominated by men but woman have more other women on their network this would explain this result.*

**Answer:** We completely agree with the reviewer. The observed results suggest that both men and women might tend to choose as keynote speakers or coauthors people that are “similar” or of the same gender to one-self (lines 387-391).

*Line 383: could this bias also be explained by the invited female speakers being less well known as the male ones? (e.g. check citations or maybe also age of the presenters) See also Maas et al 2021, DOI: 10.1111/conl.12797, on women being underrepresented in the list of top-publishing authors, thus they are less visible, and thus less desirable for keynote positions?*

**Answer:** Yes, the lower recognition of women researchers might also explain the low number of attendees. We included this reasoning in the discussion (lines 398-399).

*Lines 389-392: if women and men are both influenced by the mentioned implicit bias, how can these results agree with the idea that women are less capable of doing science than men? Should a female scientist not have a higher opinion of the capability of other women, since she is a woman and a scientist herself? The argument has merit when discussion why less women choose science as a career path than men, but I don’t think it explains the lesser attendance at talks given by female scientists if the participation of men and women in these talks was equal.*

**Answer:** While the reviewer’s rationale is reasonable, we believe that the implicit bias exists within the community of female scientists as well. Several works have shown that women are stricter in their evaluation criteria when they are evaluating other women [e.g. Moss-Racusin et al. 2012]. Also, as the reviewer mentioned, women are underrepresented in the list of top-publishing authors. Therefore, both men and women might have chosen those talks performed by top-publishing authors (i.e. men). We added this rationale in the discussion (lines 403-405).

*Line 427: self-report.*

**Answer:** Ok, changed (line 439).

*Line 435: please give an example how women are excluded from social events during this conference.*

**Answer:** Women are often indirectly excluded from social events, such as after-conference activities, because they have to reconcile attendance to conferences with domestic or familiar obligations (e.g. parenthood). This rationale has been added in the text according to the reviewer comment (line 446-448).

**References**

Bailey EG, Greenall RF, Baek DM, Morris C, Nelson N, Quirante TM, et al. Female in-class participation and performance increase with more female peers and / or a female instructor in life sciences courses. CBE—Life Sci Educ. 2020;19: ar30. doi:10.1187/cbe.19-12-0266

Biggs J, Hawley PH, Biernat M. The academic conference as a chilly climate for women: effects of gender representation on experiences of sexism, coping responses, and career intentions. Sex Roles. 2018;78: 394–408. doi:10.1007/s11199-017-0800-9

Calisi RM, Working Group of Mothers in Science. How to tackle the childcare-conference conundrum. Proc Natl Acad Sci U S A. 2018;115: 2845–2849. doi:10.1073/pnas.1803153115

Carter AJ, Croft A, Lukas D, Sandstrom GM. Women’s visibility in academic seminars: Women ask fewer questions than men. PLoS One. 2018;13: 212146. doi:10.1371/journal.pone.0202743

Ceci SJ, Ginther DK, Kahn S, Williams WM. Women in academic science: A changing landscape. Psychol Sci Public Interes. 2014;15: 75–141. doi:10.1177/1529100614541236

Chang EH, Milkman KL, Gromet DM, Rebele, RW, Massey C, Duckworth AL, Grant AM. The mixed effects of online diversity training. PNAS. 2019;116(16): 7778-7783; doi:10.1073/pnas.1816076116

Cook C, Heath F, Thompson RL. A meta-analysis of response rates in web- or internet-based surveys. Educational and Psychological Measurement. 2000;60: 821–836. doi:10.1177/00131640021970934.

Karpowitz CF, Mendelberg T, Shaker L. Gender inequality in deliberative participation. Am Polit Sci Rev. 2012;106: 533–547. doi:10.1017/S0003055412000329

King L, MacKenzie L, Tadaki M, Cannon S, McFarlane K, Reid D, et al. Diversity in geoscience: Participation, behaviour, and the division of scientific labour at a Canadian geoscience conference. Facets. 2018;3: 415–440. doi:10.1139/facets-2017-0111

Konrad AM, Corrigall E, Lieb P, Ritchie JE. Sex differences in job attribute preferences among managers and business students. Gr Organ Manag. 2000;25: 108–131. doi:10.1177/1059601100252002

L'Oréal-UNESCO. For Women in Science Survey. 2015

Moss-Racusin CA, Dovidio JF, Brescoll VL, Graham MJ, Handelsman J. Science faculty’s subtle gender biases favor male students. Proc Natl Acad Sci USA. 2012;109: 16474–9. doi:10.1073/pnas.1211286109

Poushter J, Fetterolf J, Tamir C. 2019. A Changing World: Global Views on Diversity, Gender Equality, Family Life and the Importance of Religion. Pew Research Center. 2019. <https://www.pewresearch.org/global/2019/04/22/how-people-around-the-world-view-gender-equality-in-their-countries/>

Slauson‐Blevins K, Johnson KM. Doing gender, doing surveys? Women's gatekeeping and men's non‐participation in multi‐actor reproductive surveys. *Sociological Inquiry*. 2016;*86*(3): 427-449. doi: [10.1111/soin.12122](https://doi.org/10.1111/soin.12122)

Telis N, Glassberg EC, Pritchard JK, Gunter C. Public discussion affects question asking at academic conferences. Am J Hum Genet. 2019;105: 189–197. doi:10.1016/j.ajhg.2019.06.004
